# SARS-CoV-2 antibody prevalence, titres and neutralising activity in an antenatal cohort, United Kingdom, 14 April to 15 June 2020

**DOI:** 10.2807/1560-7917.ES.2020.25.41.2001721

**Published:** 2020-10-22

**Authors:** Sheila F Lumley, David W Eyre, Anna L McNaughton, Alison Howarth, Sarah Hoosdally, Stephanie B Hatch, James Kavanagh, Kevin K Chau, Louise O Downs, Stuart Cox, Laura Dunn, Anita Justice, Susan Wareing, Kate Dingle, Justine Rudkin, Kathryn Auckland, Alexander Fyfe, Jai Bolton, Robert Paton, Alexander J Mentzer, Katie Jeffery, Monique I Andersson, Tim James, Tim E A Peto, Brian D Marsden, Gavin Screaton, Richard J Cornall, Paul Klenerman, Daniel Ebner, David I Stuart, Derrick W Crook, Nicole Stoesser, Stephen H Kennedy, Craig Thompson, Sunetra Gupta, Philippa C Matthews

**Affiliations:** 1 Nuffield Department of Medicine, University of Oxford, Medawar Building, South Parks Road, Oxford, United Kingdom; 2 Department of Microbiology/Infectious Diseases, Oxford University Hospitals NHS Foundation Trust, John Radcliffe Hospital, Oxford, United Kingdom; 3 These authors contributed equally to this work; 4 Nuffield Department of Population Health, University of Oxford, Big Data Institute, Old Road Campus, Oxford, United Kingdom; 5 Nuffield Department of Medicine, John Radcliffe Hospital, Oxford, United Kingdom; 6 Target Discovery Institute, Nuffield Department of Medicine, University of Oxford, Big Data Institute, Old Road Campus, Oxford, United Kingdom; 7 Department of Clinical Biochemistry, Oxford University Hospitals NHS Foundation Trust, John Radcliffe Hospital, Oxford, United Kingdom; 8 Department of Zoology, University of Oxford, Medawar Building, South Parks Road, Oxford, United Kingdom; 9 Kennedy Institute of Rheumatology, NDORMS, University of Oxford, Old Road Campus, Roosevelt Drive, Headington, Oxford, United Kingdom; 10 Structural Genomics Consortium, Nuffield Department of Medicine, University of Oxford, Old Road Campus Research Building, Roosevelt Drive, Headington, Oxford, United Kingdom; 11 The Division of Structural Biology, Nuffield Department of Medicine, University of Oxford, The Henry Wellcome Building, Roosevelt Dr, Headington, Oxford, United Kingdom; 12 Wellcome Centre for Human Genetics, Nuffield Department of Medicine, Roosevelt Drive, Headington, Oxford, United Kingdom; 13 Nuffield Department of Women’s & Reproductive Health, University of Oxford, John Radcliffe Hospital, Oxford, United Kingdom

**Keywords:** antenatal, pregnancy, COVID-19, antibody, IgG, serology, epidemiology, prevalence, ELISA, neutralisation

## Abstract

SARS-CoV-2 IgG screening of 1,000 antenatal serum samples in the Oxford area, United Kingdom, between 14 April and 15 June 2020, yielded a 5.3% seroprevalence, mirroring contemporaneous regional data. Among the 53 positive samples, 39 showed in vitro neutralisation activity, correlating with IgG titre (Pearson’s correlation p<0.0001). While SARS-CoV-2 seroprevalence in pregnancy cohorts could potentially inform population surveillance, clinical correlates of infection and immunity in pregnancy, and antenatal epidemiology evolution over time need further study.

During the first peak of the coronavirus disease (COVID-19) pandemic in the United Kingdom (UK) between April and June 2020, we set out to generate a benchmark estimate of antenatal severe acute respiratory syndrome coronavirus 2 (SARS-CoV-2) seroprevalence in a population of pregnant women in the Oxford area. We also evaluated an IgG enzyme-linked immunosorbent assay (ELISA) head-to-head with a pseudotyped virus neutralisation test in this group, and investigated the extent to which routine antenatal testing for SARS-CoV-2 IgG antibody could inform population surveillance efforts.

## Samples and processing pipeline

**Figure 1:**
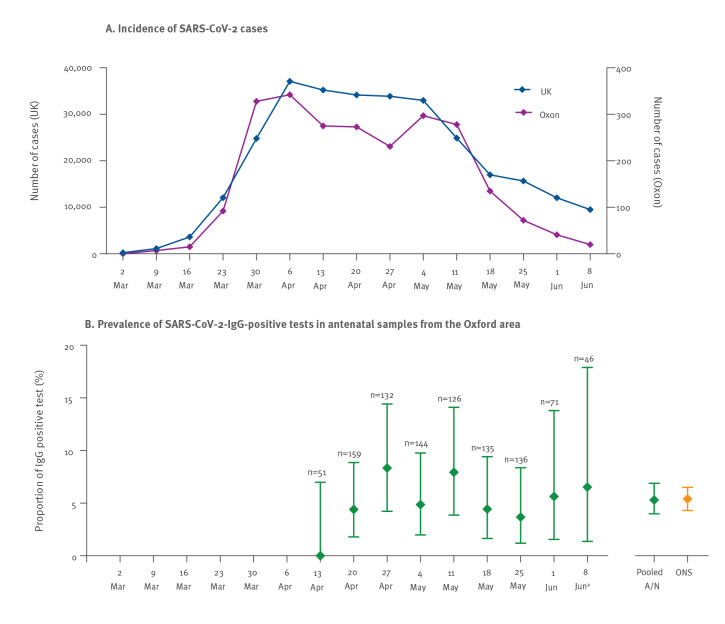
(A) Incidence of SARS-CoV-2 cases in Oxfordshire and the United Kingdom during the first peak of the COVID-19 pandemic, shown in parallel with (B) prevalence of SARS-CoV-2-IgG-positive antenatal samples from the Oxford area, presented by week, March–June 2020

We analysed antenatal serum samples that had been taken in the Oxford area, during the first trimester of pregnancy (typically at 8–12 weeks’ gestation), from women aged 17 to 48 years (median: 32 years; interquartile range (IQR): 28–35) over a period spanning 9 weeks between 14 April and 15 June 2020. This coincided with the first peak of the national and local COVID-19 pandemic (Figure 1A). Samples were collected after routine clinical laboratory tests had been completed (Figure S1). We used primary sample identifiers to obtain year of birth, self-reported ethnicity, Oxfordshire postcode district (first three or four digits), and index of multiple deprivation (IMD) from the electronic patient records (EPR). We excluded samples for which no EPR record was available, and those with a missing postcode, generating a final dataset of 1,000 consecutive samples with supporting clinical metadata. To track laboratory processing, samples were assigned a new unique barcode identifier. Aliquots were kept at 4 °C throughout and prepared for laboratory assays using a Janus liquid handler (PerkinElmer, Waltham, Massachusetts, United States) (Figure S1). For comparing results from our cohort of pregnant women, to a regional (South-East England) seroprevalence estimate during a similar period, we used data from the Office for National Statistics (ONS), which had overseen a large population serosurvey [1].

A/N: antenatal; COVID-19: coronavirus disease; ONS: Office of National Statistics data; Oxon: Oxfordshire; SARS-CoV-2: severe acute respiratory syndrome coronavirus 2.

(A) Incidence of SARS-CoV-2 cases, based on nose/throat swab reverse-transcription PCR testing; purple line shows diagnosed cases in Oxfordshire; blue line shows diagnosed cases in England (data from public sources [22,23]). (B) SARS-CoV-2 IgG prevalence in antenatal serum samples is shown in green, from the week commencing 13 April (bars show 95% confidence intervals; number at the top of each bar indicates number sampled during this week). Final two bars show pooled data for the Oxford area A/N sample collection (green), compared with data for South-East England collected by the ONS, for which SARS-CoV-2 IgG prevalence is shown for the period 26 April–8 June, measured using the same ELISA (orange) [1].

^a^ Data for the last 2 weeks of our serosurvey are pooled due to small numbers tested in the final week, where testing was only conducted on 1 day (15 June).

## ELISA for detection of IgG to SARS-CoV-2 spike protein

Samples were tested using a new 384-well ELISA established at the University of Oxford, which detects IgG to trimeric SARS-CoV-2 spike protein, with a sensitivity of 99.1% (95% confidence interval (CI): 97.8–99.7) and specificity 99.0% (95% CI: 98.1–99.5) as recently described [2,3]. The threshold for positivity in this assay is 8.0×10^6^ standard units.

## Pseudotyped virus neutralisation assays

SARS-CoV-2 pseudotyped microneutralisation (pMN) assays were undertaken using methods previously described [4,5]. Briefly, a lentivirus particle was constructed to display the full SARS-CoV-2 spike protein. Infectivity was determined by incubating the pseudovirus particle together with twofold serial dilutions of test sera with HEK-293T-ACE2-plasmid-transfected cells, generating a luciferase read out in relative light units (RLU) after incubation at 37 °C for 72 hours. The laboratory work was undertaken blinded to the results of the serology assay and to the location of positive controls on the plates.

## Ethical statement

This work was approved by the South Central Research Ethics Committee (Ref: 08/H0606/139).

## Prevalence and distribution of IgG in antenatal population

**Figure 2:**
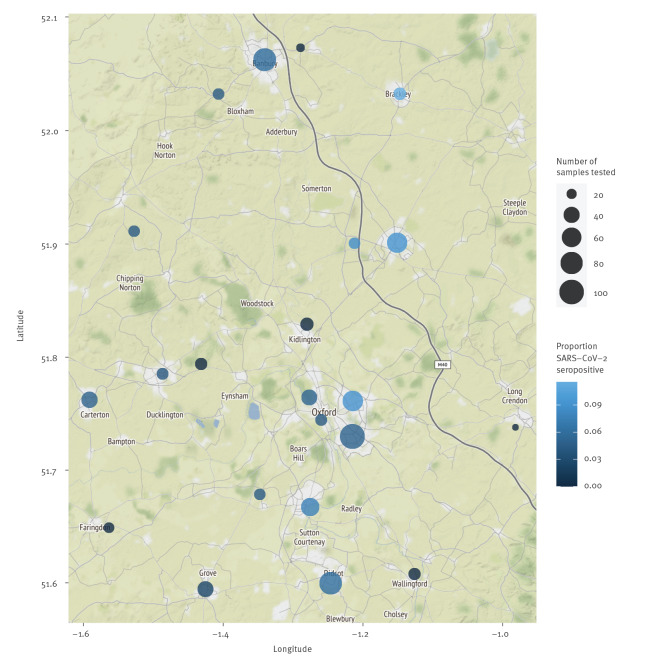
Map showing the location and prevalence of SARS-CoV-2 IgG-positive tests in antenatal women, Oxford area, South-East England, United Kingdom, March–June 2020 (n = 1,000 antenatal women tested^a^)

The overall prevalence of SARS-CoV-2 anti-spike IgG in this antenatal cohort was 53/1,000 (5.3%; 95% CI: 4.0–6.9%), which closely mirrors ONS population surveillance data for South-East England in a similar time period (26 April–8 June), at 5.4% (95%CI: 4.3–6.5%); (Figure 1). There was no observed change in prevalence by week surveyed (Figure 1B) and IgG status was not associated with maternal age (p = 0.6), self-reported ethnicity (p = 1.0), or IMD score (p = 1.0); (Figure S2). Sampling density was highest in urban areas (clustered around Oxford city centre), but there were no obvious geographical hotspots, with seropositive samples originating from across the region (Figure 2).

SARS-CoV-2: severe acute respiratory syndrome coronavirus 2.

^a^ Data are shown for postcodes in which ≥ 10 serum samples were collected.

## Quantification and interpretation of viral neutralisation

**Figure 3:**
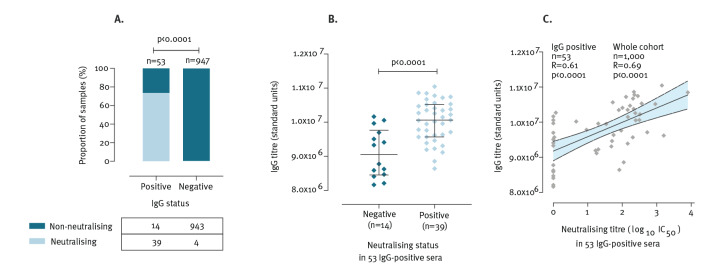
Relationship between SARS-CoV-2 IgG (standard units by ELISA) and neutralisation (based on pseudovirus microneutralisation assay) in serum samples from antenatal women in the Oxford area, United Kingdom, (n = 1,000 women tested)

CI: confidence interval; ELISA: enzyme-linked immunosorbent assay; IC50: half maximal inhibitory concentration; SARS-CoV-2: severe acute respiratory syndrome coronavirus 2.

Overall, neutralising activity was detected in 43/1,000 (4.3%) samples. Among these, 39 were IgG-positive and four IgG-negative. Thus, 39 of 53 IgG-positive sera had evidence of neutralising activity in vitro (Figure 3A). Among the IgG-positive samples, those with neutralising activity had significantly higher quantitative IgG titres than those that were non-neutralising, p < 0.0001 (Figure 3B), in keeping with previous reports [6,7]. Among the 53 IgG-positive samples, half maximal inhibitory concentration (IC_50_) in pMN was correlated with IgG concentration; for each log_10_(IC_50_) increase in neutralising activity, IgG titres were 410,000 (95%CI: 260,000–560,000) units higher (p < 0.0001, Figure 3C).

(A) Concordance between IgG status (i.e. negative or positive; threshold for positivity set at 8.0×106 standard units) and neutralising activity of serum samples (n = 1,000); p-value by Fisher’s Exact Test; (B) IgG titres in samples meeting the threshold for IgG-positivity (n = 53), grouped according to neutralisation status; lines indicate median and interquartile range; p-value by Mann–Whitney U test. Four IgG-negative samples in which neutralising activity was detected are shown in Figure S3; (C) Relationship between quantitative ELISA read-out and neutralisation expressed as log_10_ IC_50_ in IgG-positive samples (n = 53). Shaded area shows 95% CI. R and p-value by Pearson’s correlation are presented both the 53 datapoints shown, and for the whole sample set of 1,000 sera (among which those with no measurable neutralising activity were assigned as zero).

Neutralising activity suggests that the anti-spike antibodies measured by the pMN assay offer protection against infection and/or disease in the majority of samples designated seropositive by ELISA, but further clinical correlation is required. 

Interestingly, 4/947 (0.4%) SARS-CoV-2 IgG-negative samples had neutralising activity, of which three had IgG titres close to the positive threshold (Figure S3). This result highlights the stringent threshold set for IgG-positivity (to assure assay specificity), suggesting that in some cases sufficient IgG titres may be present to mediate protective immunity even when the sample is reported as seronegative. In the lowest-titre IgG-negative sample with neutralising activity (Figure S3), it is possible that cross-reactive antibodies (not detected on a specific anti-spike IgG ELISA) could be responsible for neutralisation, with corresponding protection against infection or disease; this hypothesis warrants further investigation.

## Discussion

SARS-CoV-2 antibody testing has been scaled up at pace in many countries throughout 2020 to develop insights into the epidemiology of infection and inform interventions for infection prevention and control [2,8-11]. In the UK, serum samples are collected as part of routine antenatal care, providing an opportunity for serosurveillance with fewer logistical and resource implications than other population groups [12]. There is interest in the extent to which pregnant women might represent a ‘sentinel group’, providing an accessible snapshot of SARS-CoV-2 seroprevalence in the general population. However, pregnant women represent a small part of the overall 18–40 year-old population, and although our study results suggest that seroprevalence in this group is congruent with population estimates, this observation should be extrapolated with caution and may change over time. Antenatal incidence may decline compared with other population groups, as pregnant women behave cautiously, with close observance of social distancing and other measures to reduce exposure [13]. Samples taken at 8 to 12 weeks’ gestation, when pregnant women may not yet have fully established behaviours to increase their protection, might be more relevant as a sentinel group for the wider population than serology performed at delivery, but the observation we make in the first epidemic wave may not hold true in future outbreaks.

Our sample collection represents local population density, with urban areas relatively over-represented, but – given the low seroprevalence – we were underpowered to identify associations between seropositivity and other maternal characteristics. The IMD of the region sampled is not representative of the UK, as Oxfordshire ranks 142/151 of upper tier local authority districts, putting it in the 10% least deprived areas of the UK. Other studies of SARS-CoV-2 IgG seroprevalence in pregnancy have generated higher point estimates than in our setting, for example seroprevalence was 8% in a study in France [14], 10% in Italy [15], and 14% in Spain [16]; differences may reflect timing of sample collection and local population epidemiology, as well as different performance characteristics of the assays used to measure antibodies.

Mapping the exposure of pregnant women to SARS-CoV-2 infection is important, as they represent a potentially vulnerable group [17,18]; more evidence is required to determine whether pregnancy is a risk factor either for acquisition of infection or for severe maternal disease [19,20], and to identify adverse fetal or neonatal outcomes. More focus is also needed for potentially high-risk groups (for example according to ancestry, deprivation, maternal age), and to understand the impact of changes in behaviour (and thus exposure) during pregnancy in different populations over time. Prospective surveillance through programmes such as INTERCOVID have been established to address these questions [21].

In conclusion, our study suggests that routine surveillance of SARS-CoV-2 antibody in sera collected early in pregnancy may be a useful tool for monitoring wider population IgG prevalence, but scrutiny will be required to track this observation over time.

## Supplementary Data

236000 Supplementary Material
