# Supplementary material for: SARS-CoV-2 antibody prevalence, titres and neutralising activity in an antenatal cohort, United Kingdom, 14 April to 15 June 2020
Source: Euro Surveill. 2020 Oct 22;25(42):2001721. doi: 10.2807/1560-7917.ES.2020.25.41.2001721 (PMC7651878; doi:10.2807/1560-7917.ES.2020.25.41.2001721)

SUPPLEMENTARY MATERIAL

This supplementary material is hosted by *Eurosurveillance* as supporting information alongside the article 'SARS-CoV-2 antibody prevalence, titres and neutralising activity in an antenatal cohort, United Kingdom, 14 April to 15 June 2020' on behalf of the authors, who remain responsible for the accuracy and appropriateness of the content. The same standards for ethics, copyright, attributions and permissions as for the article apply. Supplements are not edited by *Eurosurveillance* and the journal is not responsible for the maintenance of any links or email addresses provided therein.

Table S1: Metadata table for 1000 anonymised serum samples

Metadata are published on Figshare: Lumley, SF; Eyre, D McNaughton, A; Howarth, A; Hatch, S; Jeffery, K, et al. (2020): Serological survey of SARS-CoV-2 IgG in antenatal women in Oxfordshire, April-July 2020. Figshare. https://doi.org/10.6084/m9.figshare.12834641.v1

Figure S1: Flow diagram to show pathway of sample collection and processing.

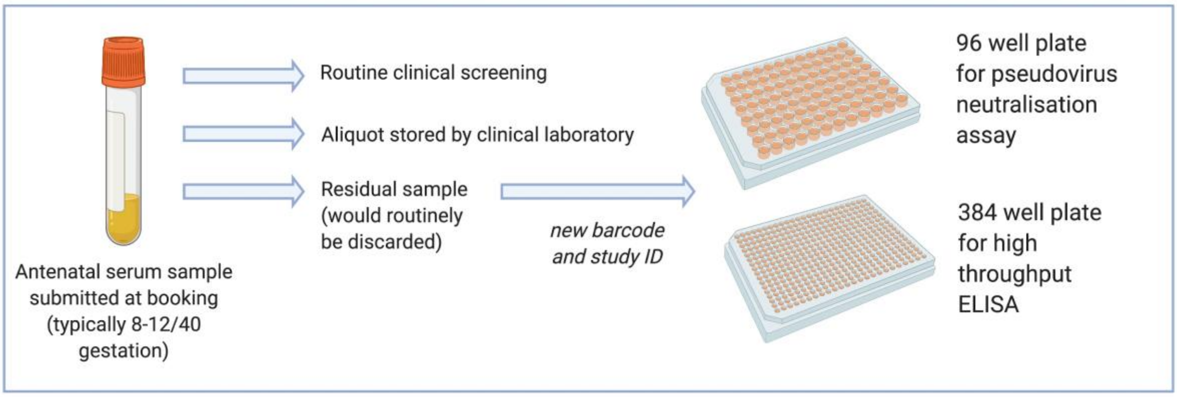

Figure S2: Relationship between demographic characteristics and SARS-CoV-2 IgG serostatus in 1000 antenatal women enrolled for maternity care in Oxfordshire. (A) IgG status vs. maternal age; boxes show median and IQR, whiskers show 5-95 percentile; p-value by Mann-Whitney U test. (B) IgG status vs. index of multiple deprivation (IMD) score, p-value by Mann-Whitney U test. (C) IgG status vs. ethnicity, p-value by ANOVA. The 'other' category includes individuals for whom ethnicity data were missing.

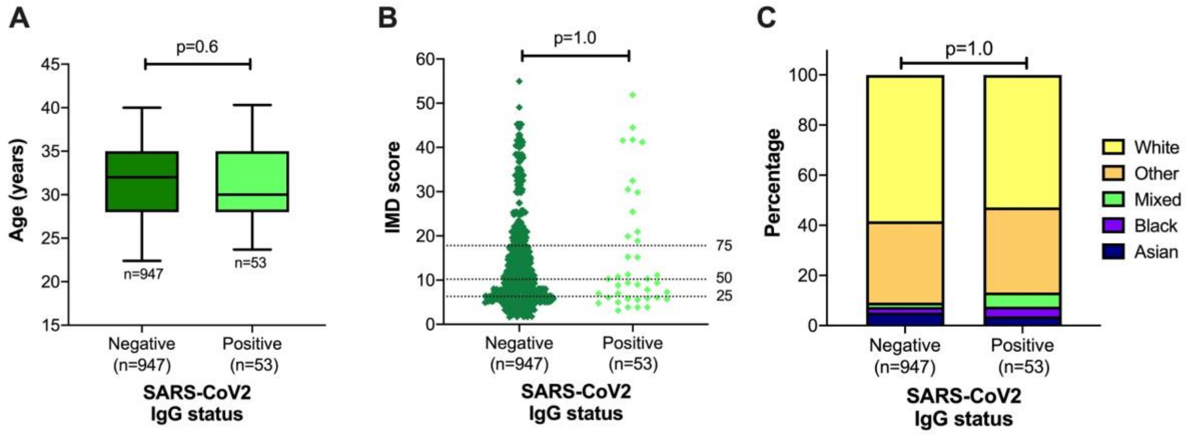

Figure S3: Relationship between *in vitro* neutralisation (based on pseudovirus microneutralisation assay) and SARS-CoV-2 IgG (standard units by ELISA) from antenatal women in the Oxfordshire area of the UK. Data are shown for 1000 consecutive samples. Dashed horizontal lines indicate threshold for IgG-positivity, set at 8.0×10^6 standard units. Among four samples with neutralising activity that were reported as IgG-negative (light blue points below the dashed line), IgG titres were 7.9×10^6, 6.9×10^6, 6.0×10^6 and 2.9×10^6 standard units.

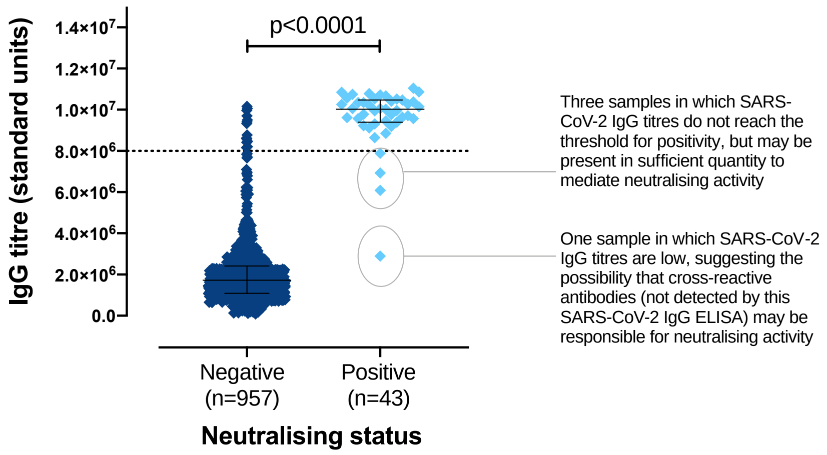

Supplement: Supplementary Material [file 2001721_Supplement.pdf]
